# 3dRS, a Web-Based Tool to Share Interactive Representations of 3D Biomolecular Structures and Molecular Dynamics Trajectories

**DOI:** 10.3389/fmolb.2021.726232

**Published:** 2021-08-13

**Authors:** Genís Bayarri, Adam Hospital, Modesto Orozco

**Affiliations:** ^1^ Institute for Research in Biomedicine (IRB Barcelona), The Barcelona Institute of Science and Technology (BIST), Barcelona, Spain; ^2^ Departament de Bioquímica i Biomedicina, Facultat de Biologia, Universitat de Barcelona, Barcelona, Spain

**Keywords:** 3D representation, biomolecular structures, data sharing, molecular dynamics, interactive figures, FAIR

## Abstract

3D Representation Sharing (3dRS) is a web-based tool designed to share biomolecular structure representations, including 4D ensembles derived from Molecular Dynamics (MD) trajectories. The server offers a team working in different locations a single URL to share and discuss structural data in an interactive fashion, with the possibility to use it as a live figure for scientific papers. The web tool allows an easy upload of structures and trajectories in different formats. The 3D representation, powered by NGL viewer, offers an interactive display with smooth visualization in modern web browsers. Multiple structures can be loaded and superposed in the same scene. 1D sequences from the loaded structures are presented and linked to the 3D representation. Multiple, pre-defined 3D molecular representations are available. The powerful NGL selection syntax allows the definition of molecular regions that can be then displayed using different representations. Important descriptors such as distances or interactions can be easily added into the representation. Trajectory frames can be explored using a common video player control panel. Trajectories are efficiently stored and transferred to the NGL viewer thanks to an MDsrv-based data streaming. The server design offers all functionalities in one single web page, with a curated user experience, involving a minimum learning curve. Extended documentation is available, including a gallery with a collection of scenes. The server requires no registration and is available at https://mmb.irbbarcelona.org/3dRS.

## Introduction

Since Max Perutz and colleagues solved the first 3D structure of Hemoglobin using X-ray crystallography in 1960 (Perutz et al., 1960), macromolecular structure 3D representations have become classic elements in biomolecular scientific journals and scientific discussions. The constant exponential growth in the number of experimentally solved structures deposited in the Protein Data Bank database (Berman et al., 2000) (PDB) and the spectacular increase in the reliability of Molecular Dynamics (MD) simulations (Hospital et al., 2015), Virtual Screening (VS) techniques (Walters and Wang, 2020) or protein structure predictions (Senior et al., 2020; Zhu et al., 2021) generates the need to deal in an efficient way with structures (3D) and structural ensembles (4D models, with time as a 4^th^ dimension). The still dominant use of flat figures or predetermined videos is hampering discussion and full exploitation of the power of structural data.

Informatics technology has evolved even faster than structural biology. Thus, current network bandwidths allow streaming of heavy videos at home, Central and Graphics Processing Units (CPUs, GPUs) are powerful enough to work with extremely demanding tasks, even in portable and small devices, and graphic software has improved dramatically their performance for realistic representations of the structural models. Some of these technologies (such as WebGL) have been already used for the representation of 3D structures in a common web browser (Rego and Koes, 2015; Bekker et al., 2016; Sehnal et al., 2017; Shi et al., 2017; Perkel, 2018; Rose et al., 2018; Hildebrand et al., 2019; Sehnal et al., 2021) using native support for GPU acceleration. Web applications and online platforms have already integrated these tools, starting with the PDB databank, which offers the possibility to represent the uploaded structures with NGL (Rose et al., 2018), Mol* (Sehnal et al., 2021) or JSMol. However, representation of MD simulation data is not as common. Although tools to automate the generation of MD videos exist (Johnson et al., 2011; Andrei et al., 2012; Wieczór et al., 2020), they lack interactivity. In terms of web-based interactive visualizers, only some of them are able to read MD trajectory data (e.g. JSMol, NGL), and are restricted to a few trajectory formats. A small number of servers are integrating MD data visualization with different degrees of interactivity (Meyer et al., 2010; Hospital et al., 2016; Rodríguez-Espigares et al., 2020; Zivanovic et al., 2020). Luckily, tools such as MDsrv (Tiemann et al., 2017) and HTMol (Carrillo-Tripp et al., 2018) came recently into play to facilitate this task, allowing streaming and visualization of MD trajectories. MDsrv accepts many different trajectory formats, thanks to the help of MDTraj (McGibbon et al., 2015) and MDAnalysis (Michaud-Agrawal et al., 2011) software packages.

However, despite all these technological advances, the practical use of 3D/4D structural images still implies visualization in a single computer, which means that interactivity with the structure is limited to a handful of scientists located in a particular laboratory. The inability to interact with 3D/4D structures is especially annoying when dealing with published material. Thus, conference proceedings and journals provide electronic copies of the papers, including pictures or in some cases videos of the structures. However, interactive tools are rarely used (Perkel, 2018) (e.g. https://www.elsevier.com/authors/tools-and-resources/data-visualization), (https://aasnova.org/2021/02/26/aas-publishing-news-interactive-figures), and have hardly been applied in the context of biomolecular structural information. As a result, the reader of a scientific paper has to rely on the interpretation of the author of a flat image, or alternatively, retrieve the structure from PDB and redo the imaging in a process that might take hours or even days.

Pioneering efforts towards figure interactivity have been done by different groups and consortia. The *Interactive 3D Complements* (I3DC) from Proteopedia (Hodis et al., 2008) was a step forward for the incorporation of interactive structure visualizations as journal figures. POLYVIEW-3D (Porollo and Meller, 2007) offered a way to create publication quality structure renders, as well as animated images (GIF) to be used as dynamic figures, and the POLYVIEW-MM (Porollo and Meller, 2010) extension included the possibility to upload molecular simulation data, generating a combination of interactive visualization with structural analysis. Web3Dmol (Shi et al., 2017) was designed to be both an interactive protein structure visualizer and a tool to share interactive representations, being the first one integrating 1D sequence, measurement tools and meta-information. More recently, "I see in 3D" (iCin3D) offered a powerful macromolecular visualizer and editor, able to synchronize the display of 3D structure, 2D interaction, 1D sequences and annotations (Wang et al., 2020). The tool is integrated in the NCBI portfolio of services, and directly connected to the Vector Alignment Search Tool (VAST) (Berger et al., 2018) and ClinVar (Landrum et al., 2018) genomic variations database. In a similar way, ProSAT+ (Stank et al., 2016) and 3DBionotes (Tabas-Madrid et al., 2016) integrate biological annotations and structural information of proteins from multiple sources. 3DBionotes includes mutations, protein-protein interactions, post-translational modifications (Segura et al., 2017) and most recently also genomic variations (Segura et al., 2019) and SARS-CoV-2 related annotations (Macias et al., 2021). Although the main goal of these tools is to extract evidence relating sequence, structure and function using the connection with external databases annotations, they all also offer the possibility to save the representation in a public URL.

3dRS wants to contribute to the effort started by these projects and create an interactive environment favoring a full exploitation of the structural information of macromolecules. It is created from our experience in discussing structural data between remote centers and from the problems we experience when reading flat structural pictures. 3dRS offers an easy and intuitive web-based Graphical User Interface (GUI), with a curated user experience, to generate and share interactive macromolecular 3D representations. 3dRS can render more than one structure at the same time. Dynamic representations from MD trajectories can be incorporated thanks to the integrated MDsrv module. The generated scene can be shared with a permanent link, contributing to the FAIR guiding principles for data management (Wilkinson et al., 2016). Besides, the shared representations can be further modified and/or expanded, using a fork process, following the procedure used by the GitHub software development and version control repository. The 3dRS server has a very intuitive use, no registration is required and is freely available at https://mmb.irbbarcelona.org/3dRS.

## Methods

**FIGURE 1:**
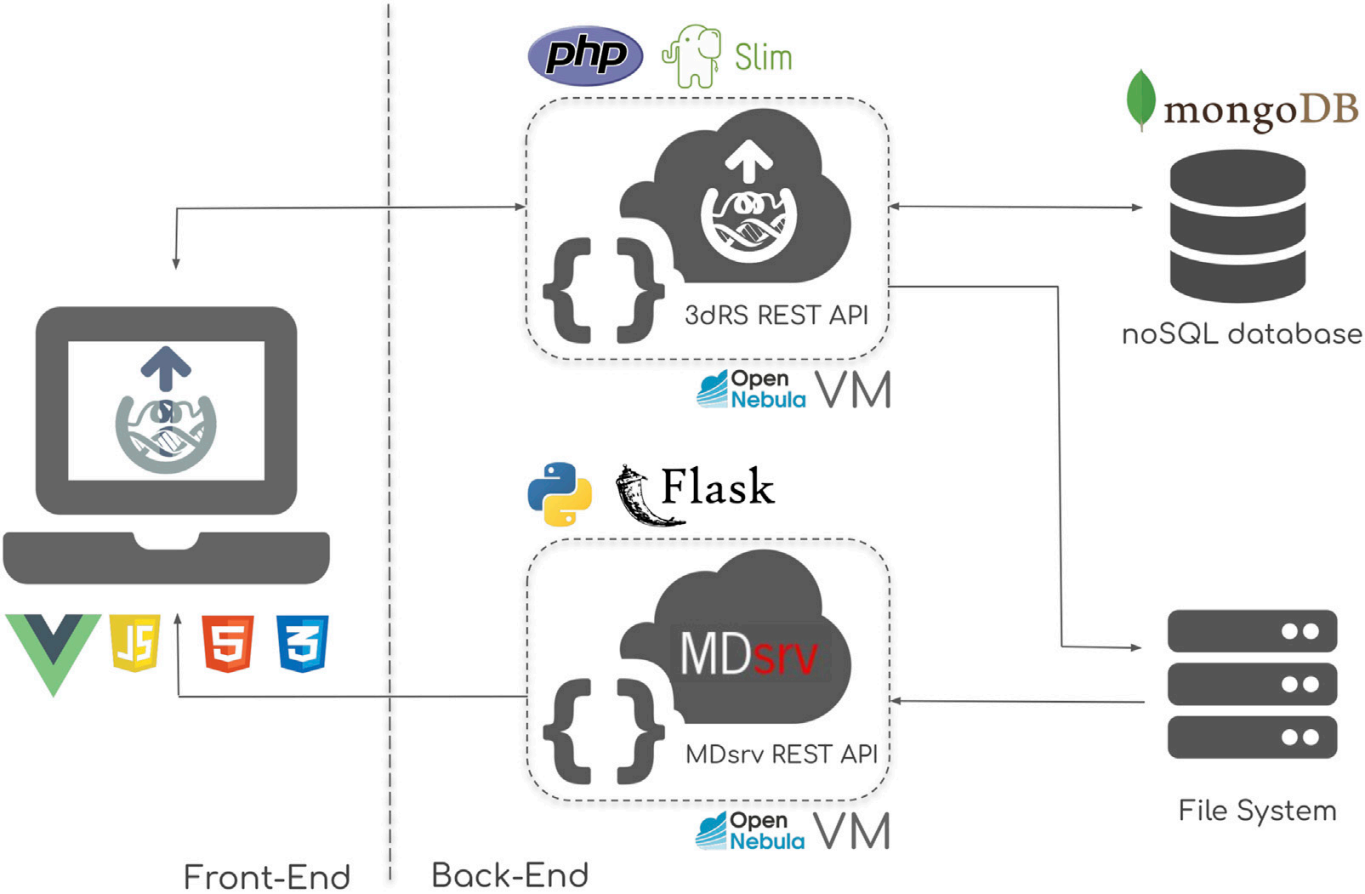
3D Representation Sharing infrastructure scheme, composed of two main sections: the front-end, powered by a combination of VueJS, HTML5 and CSS3; and the back-end, powered by a combination of PHP-Slim REST API, MDsrv MD trajectory streaming server, and mongoDB NoSQL database. The back-end tools are implemented in Virtual Machines (VM) deployed in an OpenNebula cloud infrastructure.

The server is divided in two main blocks: the front-end, embodied by the online accessible web server; and the back-end, where all data, including the representation, structures and trajectories are stored and queried through REST APIs (Figure 1).

### Front-End

The front-end implements the web-based Graphical User Interface (GUI) offered to the end user (Figure 1, left). This section includes the central part of the tool, i.e. the macromolecular structure representation routines, powered by the NGL viewer. VueJS, an open-source JavaScript framework is used for the central unit of the GUI. HTML5 and CSS3 technologies accompanying NGL and VueJS are used to polish up the final design. The result is a seamless user experience, mimicking that of a native, standalone program, without page reloads or saving buttons.

### Back-End

The back-end of the infrastructure is divided into four main parts (Figure 1, right):

- 3dRS REST API: the core programmatic interface of the platform, written using the PHP Slim micro framework. This API is the responsible for communicating the web server (user actions) with the internal database and file system.
- MDsrv REST API: in charge of streaming MD trajectories data from the file system to the web-based interface. Written in Python Flask micro framework.
- mongoDB database: is the heart of the API, where all the representations created and structures uploaded by the end users are efficiently stored.
- File system: used for storing uploaded MD trajectories.

All the important information is automatically saved to the database using the 3dRS REST API, in a transparent manner.

## Modes

3dRS offers an easy, web-based 3D macromolecular representation generator that can be then shared and reproduced with just a URL. Thus, the tool is divided in these two main working modes: *edition* and *shared*. The *edition mode* is used to add representations to the molecule(s). Such representations can be placed by selecting molecular regions and applying NGL drawing styles. Additional information such as measures (distances, angles) or labels can also be included. Once the final representation is ready, it can be shared through a permanent link. The scene is shared as it is shown in the editor, keeping not just the molecular representations, but also the orientation, zoom, background color, measures, and labels (if added). The permanent link opens the *shared mode*, which allows interactive exploration of the scene, hiding the representation menus. If the author of the scene has enabled forking, the project can be cloned, opening the *edition mode* again and recovering all the representations, which allows an easy creation of new scenes by just modifications on top of already existing projects.

### Edition Mode

**FIGURE 2:**
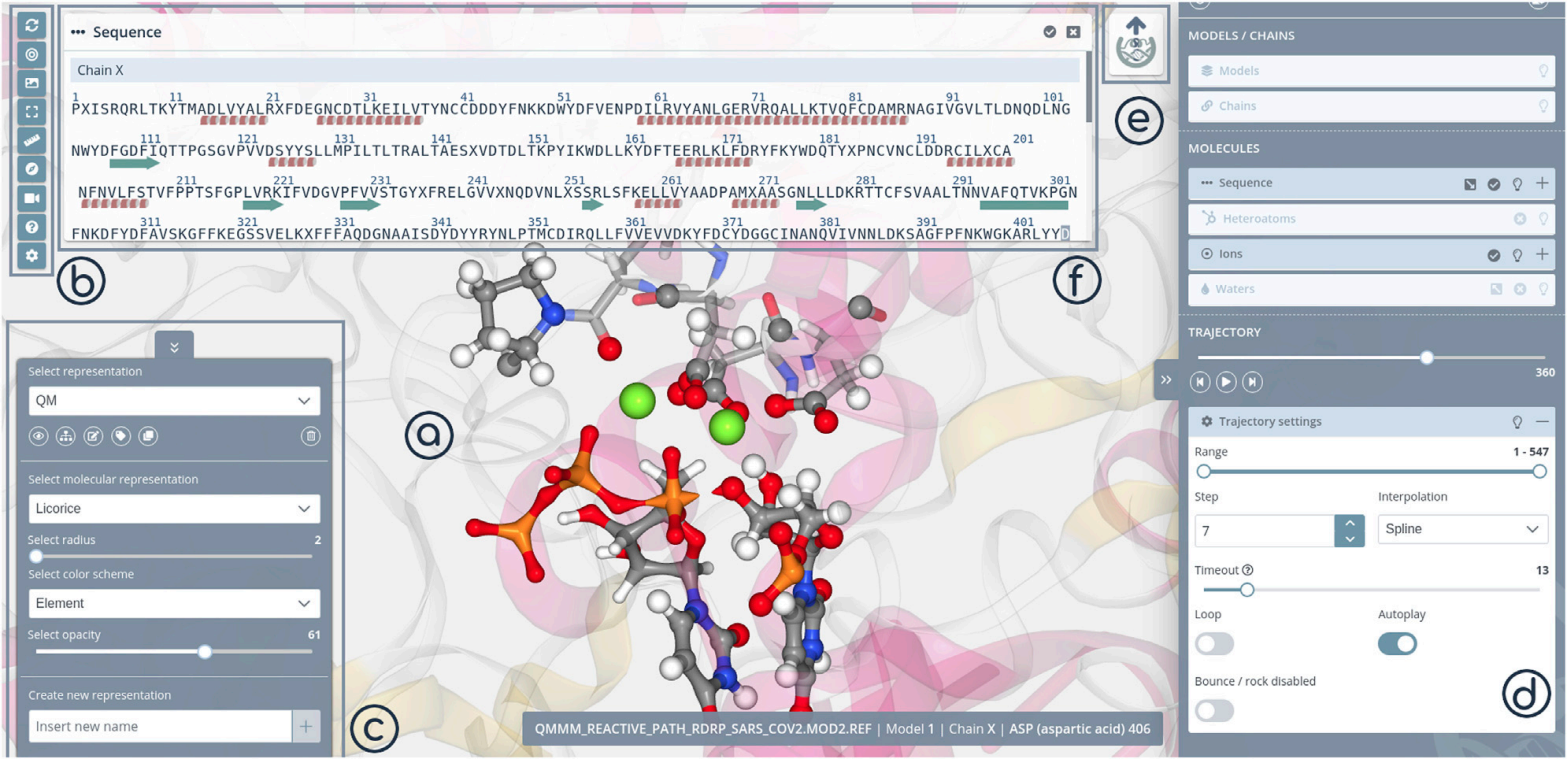
3dRS Edition Mode panels. **(A)** Stage panel; **(B)** Tools; **(C)** Representations; **(D)** Selections; **(E)** Sharing; **(F)** 1D Sequence.

3dRS edition mode is automatically opened after uploading one or more structures. It allows the creation of new representations with a live and interactive view of the structure(s) and the changes made in the central panel (Stage, Figure 2A). Visualization tools and project settings are accessible from a sidebar (Tools, Figure 2B). Available tools and settings vary depending on the working mode (edition/shared). New representations are added from a specific menu (Select Representation, Figure 2C). New representations are always linked to selections on the molecule(s), which are generated with a different specific menu (Selections, Figure 2D). The final representation can be shared clicking at the 3dRS logo button (Ready to share, Figure 2E).

The selection of the macromolecular regions to apply different drawing styles in 3dRS is inspired by the Visual Molecular Dynamics (VMD) tool (Humphrey et al., 1996), and is particularly powerful. Residue/Nucleotide sequences are opened in a new modal window (Figure 2F), which is synchronized with the 3D representation and the selection panel (Figures 2A,D). Pointing the mouse on a determined residue in the sequence window will highlight this residue in the representation and in the selection panel, and the other way around. Clicking on this residue will add it into the current selection. Segments of the sequence can be easily selected. To increase the selection power, a custom/manual selector is added, for advanced users to be able to use NGL syntax to generate complex selections.

### Shared Mode

**FIGURE 3:**
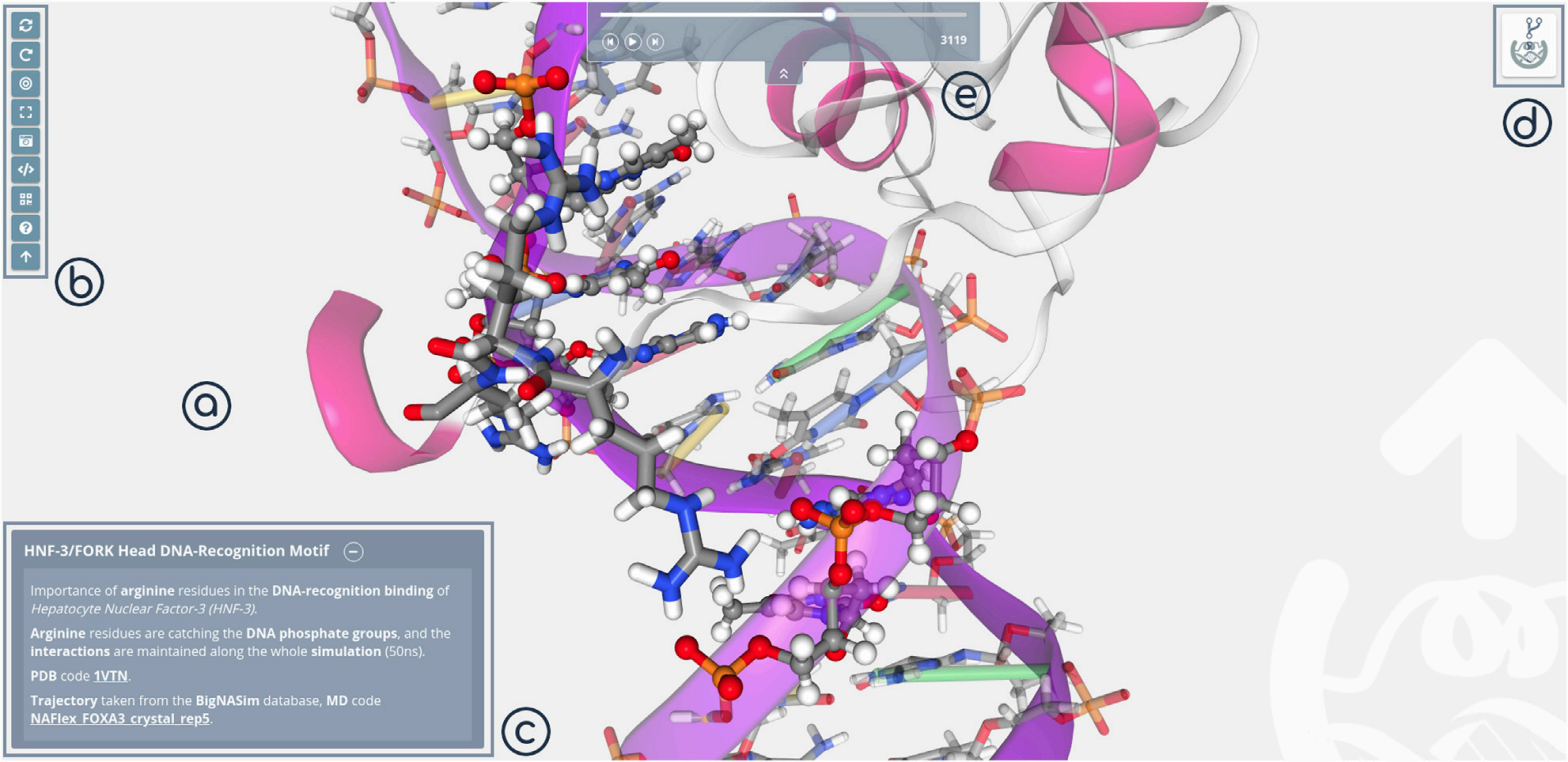
3dRS Shared Mode panels. **(A)** Stage panel; **(B)** Tools; **(C)** Caption; **(D)** Forking; **(E)** Player.

3dRS shared mode is opened by default when using 3dRS sharing links. It presents an interactive figure, a representation of a macromolecular 3D structure that can be explored in 3D, with the possibility to rotate, translate, zoom in/out and identify the atoms/residues/chains by just hovering on top of the particular molecular regions. Stage (Figure 3A) and Tools/Settings (Figure 3B) panels are maintained, with the tools bar offering different features in this case (e.g. screenshot, embed representation, QR code). Edition panels (Representations, Selections) are substituted here by the figure caption (Figure 3C), which works exactly as the captions in scientific journals, with the advantages of the web, adding rich text and including external links. Finally, the sharing logo button has been substituted by the Forking button (Figure 3D), giving the possibility to clone the project to either extend or modify it. This feature is only available if the original author of the representation has enabled forking on the project. In case of sharing a structure and a trajectory, an easy player is shown on the top of the screen (Figure 3E). This player offers the ability of controlling the given trajectory in a simple way (play/pause, move frame by frame back and forward and jump to a concrete frame through the slider bar).

## Results

3dRS web platform allows the generation of shareable interactive 3D representations from a very easy and intuitive interface. It can load structures directly from the PDB databank (PDB code) or from disk (PDB file). Multiple structures can be submitted at once, with the possibility to superpose them once loaded. MD trajectory data can be inserted for each of the structures in the scene, with an efficient streaming management thanks to the integrated MDsrv tool. 3dRS is compatible with PDB and GRO input structure files formats, with a size limit of 50 MB. For the trajectory data, XTC, DCD, TRR, BINPOS, and NETCDF formats are accepted, with a file size limit of 500 MB. Measures (distances, angles) and labels can be easily inserted in the scene. The whole representation can be shared with just a permanent URL link, with the possibility to extend or modify the representation, forking the project.

3dRS follows FAIR data guiding principles (Wilkinson et al., 2016). Representations have a globally unique and persistent identifier (permanent link). They are accessible using a standardized communications protocol (web browser). Metadata included in the representation can be used to determine the structure provenance (PDB, local file) and to further reproduce and extend the generated scene (fork). FAIR principles have been also applied in the software development process (Lamprecht et al., 2019). Accordingly, source code is available from GitHub repositories. The tool is registered in the bio.tools bioinformatics and Life Sciences registry (Ison et al., 2016) (https://bio.tools/3drs). Extended documentation is available from ReadTheDocs, a domain-level community standard. All the software is licensed under the Apache 2.0 license.

Examples of what 3dRS could offer to the scientific community can be found in the project gallery (https://mmb.irbbarcelona.org/3dRS/gallery). Three of these examples have been selected to demonstrate the power of the tool and are described in the following sections.

### Example 1—SARS-CoV-2 Receptor Binding Domain (RBD) Variants

**FIGURE 4:**
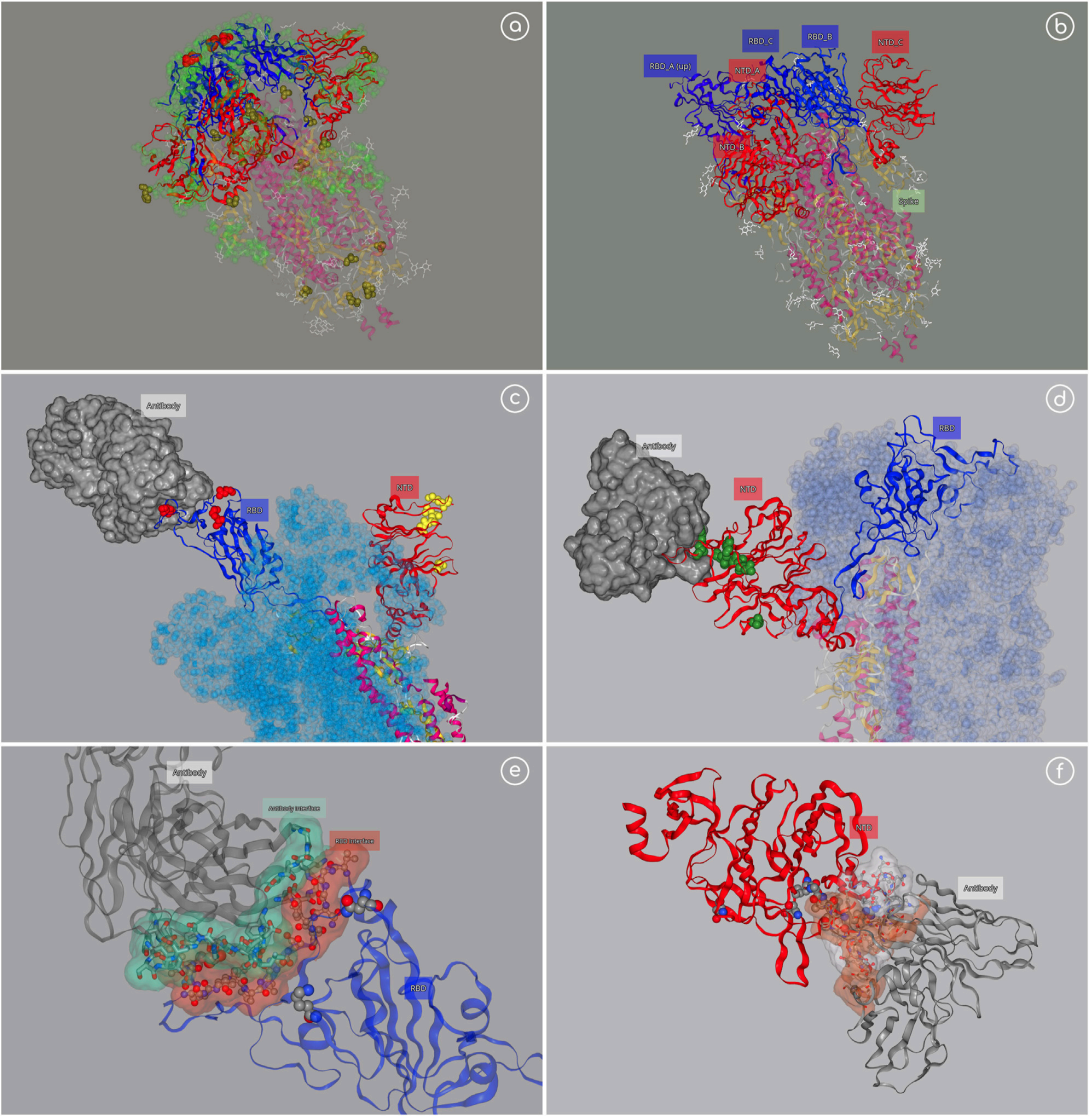
SARS-CoV-2 example. **(A)** Spike trimeric protein; https://mmb.irbbarcelona.org/3dRS/s/c1tOez; **(B)** Spike with one RDB domain in “up” position; https://mmb.irbbarcelona.org/3dRS/s/hfS2rW; **(C)** Spike (monomer) with P5A-1B9 Fab antibody attached to the RBD domain; https://mmb.irbbarcelona.org/3dRS/s/lQ6f21; **(D)** Spike (monomer) with 1–87 antibody attached to the NTD domain; https://mmb.irbbarcelona.org/3dRS/s/hJxjZv **(E)** Insight of RBD-Antibody interface; https://mmb.irbbarcelona.org/3dRS/s/JcshuF **(F)** Insight of NTD-Antibody interface; https://mmb.irbbarcelona.org/3dRS/s/YiGhxv.

Scientific groups around the world are devoting their efforts in the study of the SARS-CoV-2 and particularly in the Spike protein, a trimeric protein crucial in the virus infection and target for most of the current vaccines. Hundreds of studies directed to this protein are now available, a big part focusing on the different variants lately discovered and their impact in infectivity and vaccination resistance. Representations of the trimeric protein with the variants and known associated mutations/deletions occurring in the same lineage allow a quick identification of the location of the variants with respect to the main spike domains, with particular interest on the Receptor Binding Domain (RBD) and N-Terminal Domain (NTD), both known to contain epitopes and thus targeted regions of antibodies. 3dRS was used to generate a collection of interactive SARS-CoV-2 representations, from the complete Spike protein to the insights of the recognition interface between RBD and the human Angiotensin Converting Enzyme 2 (hACE2) (Figure 4).

Figure 4A (https://mmb.irbbarcelona.org/3dRS/s/c1tOez) and Figure 4B (https://mmb.irbbarcelona.org/3dRS/s/hfS2rW) show the trimeric Spike protein representation, with the most important RBD variants highlighted in red, associated mutations/deletions in yellow, and epitopes in green. RBD region is displayed in blue color, whereas the NTD domain is displayed in red color. Figure 4B is showing a particular Spike conformation with one RBD region in “up” position, already detached from the main trimer and prepared to recognize the human cell. Figure 4C (https://mmb.irbbarcelona.org/3dRS/s/lQ6f21) and Figure 4D (https://mmb.irbbarcelona.org/3dRS/s/hJxjZv) show a particular monomeric structure from the Spike trimer with the RBD in “up” position, attached to the antibody P5A-1B9 Fab (PDB code 7CZX) (Figure 4C) and with the NTD domain attached to the antibody 1–87 (PDB code 7L2D) (Figure 4D). Finally, Figure 4E (https://mmb.irbbarcelona.org/3dRS/s/JcshuF) and Figure 4F (https://mmb.irbbarcelona.org/3dRS/s/YiGhxv) represent an insight on the interaction regions between the RBD/NTD and the antibodies.

### Example 2—SARS-CoV-2 RNA-dependent RNA Polymerase

**FIGURE 5:**
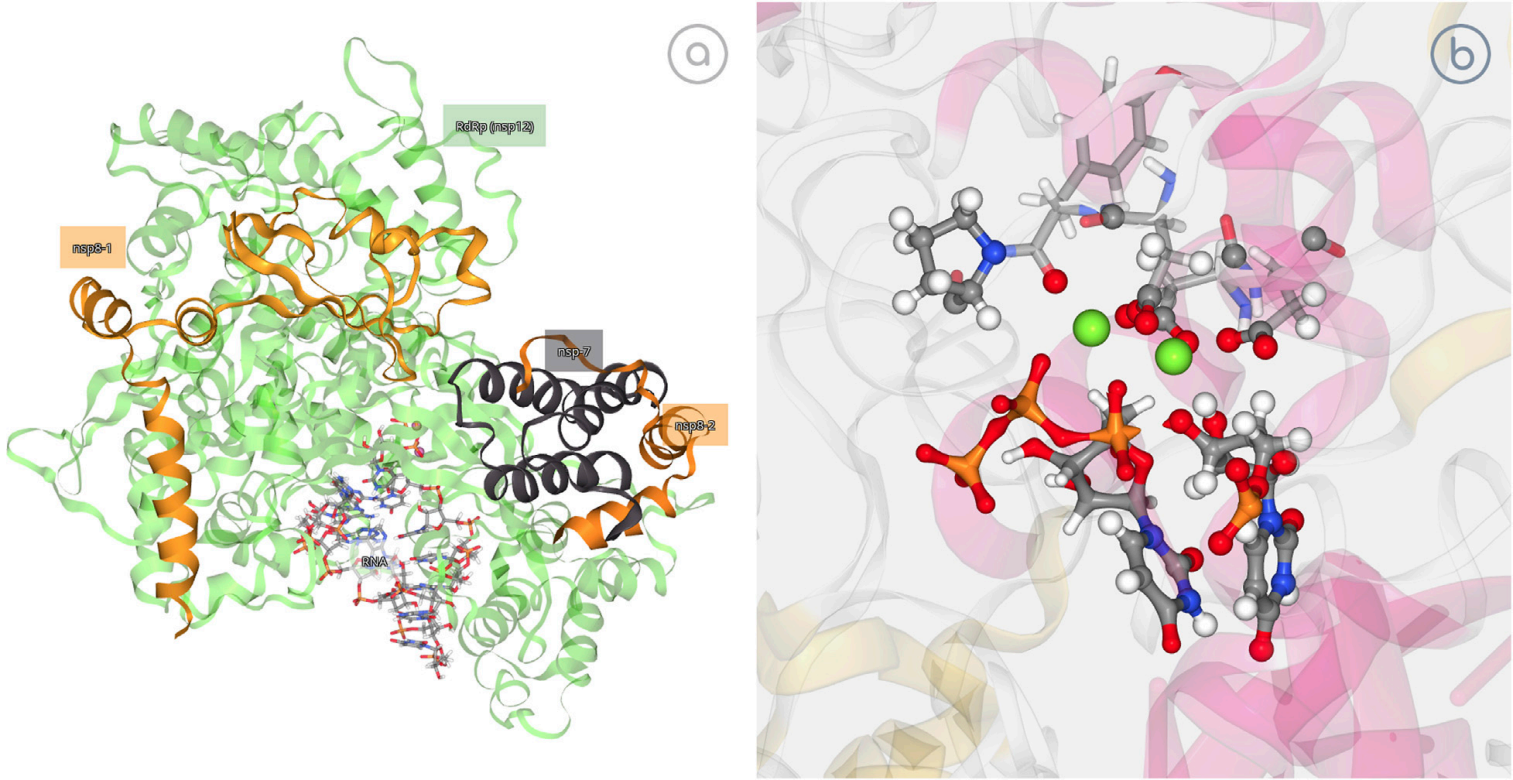
SARS-CoV-2 RNA-Dependent RNA Polymerase (RdRp) study. **(A)** Interactive representation of RdRp bound to its essential co-factors nsp7 and nsp8; https://mmb.irbbarcelona.org/3dRS/s/7Rn2Ka; **(B)** Exploring the ability of RdRp to incorporate a natural triphosphate into a nascent RNA; https://mmb.irbbarcelona.org/3dRS/s/TKf8yA.

SARS-CoV-2 RNA-dependent RNA polymerase (RdRp) is the main protein for virus replication after binding to non-structural proteins (nsp) cofactors nsp7 and nsp8 to form an active complex (Kirchdoerfer and Ward, 2019). Recent studies in the group (Aranda and Orozco, 2020) have shown that the RdRp reaction process is able to select the entering nucleotide and to catalyze its addition to a nascent RNA, following a mechanism that is similar to that of bacterial or eukaryotic polymerases with the transferred phosphate being stabilized by two Mg^2+^ ions coordinated by acidic residues of the catalytic site, while the phosphates of the incoming nucleotide being stabilized by a network of basic residues (Aranda and Orozco, 2020). In this example, the possibility of 3dRS to also represent breaking or formation of bonds as studied by QM/MM methods is showcased. 3dRS was used to generate first an interactive figure of the SARS-CoV-2 RdRp in which the attached cofactors and nucleotides are highlighted, introducing the 3D structure. Then, the process of incorporation of a natural triphosphate into a nascent RNA, exemplified by the breaking of a P1-O3P bond and the following formation of the P1-O3’ bond, is represented in an interactive and dynamic figure (Figure 5).

Figure 5A (https://mmb.irbbarcelona.org/3dRS/s/7Rn2Ka) and Figure 5B (https://mmb.irbbarcelona.org/3dRS/s/TKf8yA) show the SARS-CoV-2 RdRp representations. Figure 5A is introducing the SARS-CoV-2 RNA-Dependent RNA Polymerase bound to its essential co-factors nsp7 and nsp8, whereas Figure 5B is representing the reaction process involving a bond breaking and a new bond formation.

### Example 3—Collective Variables From Coarse-Grained Conformational Transitions

**FIGURE 6:**
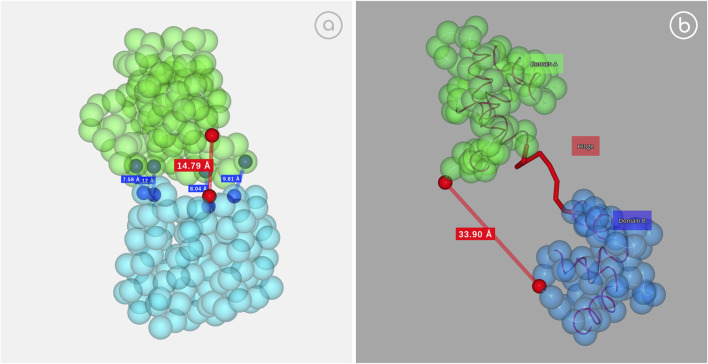
Collective variables example. **(A)** D-Ribose binding protein; https://mmb.irbbarcelona.org/3dRS/s/nDM5sO; **(B)** Calcium Saturated Cardiac Troponin C; https://mmb.irbbarcelona.org/3dRS/s/QGAgYO.

Large protein conformational changes are difficult to study experimentally and often not accessible for accurate atomistic models, forcing the use of Coarse-Grained Molecular Dynamics algorithms (Orozco et al., 2011). As an example, GOdMD method (Sfriso et al., 2013) can be used to extract relevant biophysical information to rationalize the protein motion, including a robust set of collective variables (CVs) capturing the conformational change. These CVs can then be used in more expensive and accurate calculations to refine the representation of the conformational transition. 3dRS allows the interactive and dynamic representation of some conformational transitions, highlighting the captured collective variables and showing the transition from trajectory data (Figure 6).

Figure 6A (https://mmb.irbbarcelona.org/3dRS/s/nDM5sO) and Figure 6B (https://mmb.irbbarcelona.org/3dRS/s/QGAgYO) show the conformational transitions computed with the GOdMD package for the D-ribose binding protein (Figure 6A) and the Calcium Saturated Cardiac Troponin C (Figure 6B), highlighting the collective variables with red dots. Distance between the atoms contained in the collective variables is shown as a reference.

## Discussion

3D Representation Sharing platform is a demonstration of the technical revolution in the biomolecular simulation field. Illustrations of biomolecular structures in scientific journals and conference proceeding linked for years to static, 2D figures, can be finally changed for living, interactive, 3-dimensional representations. This change, considered futuristic just 10 years ago, is now possible thanks to the fast evolution of the technology, with high-speed data transfer, greater connection bandwidths, efficient data storage tools as offered by NoSQL databases, and biomolecular visualizers able to efficiently run in common web browsers and multiple platforms and take advantage of new generation GPUs.

3dRS emerge from our experience in discussing structural models in the context of large international consortia, such as BioExcel Centre of Excellence (BioExcel CoE), ELIXIR, or Human Brain Project (HBP), where standard imaging techniques are not able to provide the desired level of interactivity between the image and the groups, based in different countries. Although an image of the structure with measurements and labels gives a first glance of these important features, it is inevitable for a scientist to wonder what is happening just behind or around the interaction, unconsciously thinking in three dimensions. And this reaction is even stronger if the picture is presenting information coming from an MD flexibility analysis, where specific molecular regions are changing depending on the frame analyzed. Interactive figures created with 3dRS are making this possible.

3dRS is to our knowledge, the only available tool making the sharing of interactive and dynamic 3D/4D custom representations possible. It keeps the process as simple as possible, easing the representation generation to reach a broader scientific community, including that with a limited knowledge on recent technologies. Generated representations are not linked to any kind of analysis or external databases as found in iCn3D or 3DBionotes platforms. The scene is shown and shared as the author conceived it, facilitating the author to highlight the details of importance in the global structure, hiding or giving transparency to less important regions of the structure, making it ideal for scientific discussion.

Quite unique of 3dRS, and also a consequence of our involvement in international consortia massively using atomistic simulations is the possibility to share MD trajectory data. Although limited in size by the web technology, the possibility to include this dynamic data is hugely increasing the value of the final representation. A small number of web-based servers can present 3D-interactive MD trajectories so far, like BigNASim (Hospital et al., 2016), or GPCRmd databases (Rodríguez-Espigares et al., 2020) (the latter relying on MDsrv and NGL tools as 3dRS), but still most dynamic information is kept and unshared in local computers. The presented platforms are designed to specifically work with local MD databases. 3dRS is opening these technologies so that the whole community can build and share dynamic figures representing MD trajectories.

3dRS software is open source (Apache2 license) and can be found in: https://github.com/gbayarri?tab=repositories&q=3drs. Documentation is written using ReadTheDocs technology, and is available from this link: https://3drs-documentation.readthedocs.io/en/latest/. The software is platform independent. It is a web-based tool, working in the most common Internet browser tools, although Google Chrome browser is recommended. The front-end is written as a Single Page Application (SPA) using VueJS, HTML5 and CSS3 technologies. The back-end is written with the PHP-Slim framework, with the MongoDB PHP Driver connecting to the underlying database, and the MDsrv server streaming MD data.

## Data Availability

The original contributions presented in the study are publicly available. This data can be found here: https://github.com/gbayarri?tab=repositories&q=3drs.

## Abbreviations

PDB, Protein Data Bank; MD, Molecular Dynamics; VM, Virtual Machine; SPA, Single Page Application; VS, Virtual Screening; CPU, Central Processing Unit; GPU, Graphics Processing Unit; WebGL, Web Graphics Library; PDF, Portable Document Format; API, Application Programming Language; HBP, Human Brain Project;
